# Supplementary material for: Healthcare Resource Consumption and Related Costs in Patients on Antiretroviral Therapies: Findings from Real-World Data in Italy
Source: Int J Environ Res Public Health. 2023 Feb 21;20(5):3789. doi: 10.3390/ijerph20053789 (PMC10000772; doi:10.3390/ijerph20053789)
Supplement: Supplementary file 1 [file ijerph-20-03789-s001.zip › ijerph-2221892-supplementary.pdf]

Supplementary materials

Table S1. Codes used for comorbidities identification.

Comorbidity	Codes
Depression	at least one prescription for ATC code: N06A
Respiratory disease	at least one prescription for ATC code: R03 or at least a hospitalization with ICD-9-CM code: 460-519
Renal failure	at least a hospitalization with ICD-9-CM code: 584-586
Alcohol/drugs abuse	at least a hospitalization with ICD-9-CM code: 303-304 or active exemption code 014
Cardiovascular disease	at least a hospitalization with ICD-9-CM code: 410-414, 431, 430, 432-438, 440, 443
Diabetes	at least one prescription for ATC code: A10
Dyslipidemia	at least one prescription for ATC code: C10
HBV/HCV	at least a hospitalization with ICD-9-CM code: 070.2, 070.3, 070.7 or active exemption code 016
Hypertension	at least one prescription for ATC code: C02, C03, C07, C08, C09

Table S2. Healthcare resources consumption in patients with TAF-based regimen during first year of follow-up by: **(A)** year of inclusion (2017-2018-2019), **(B)** persistence, **(C)** adherence (PDC >95%) and **(D)** non-adherence (PDC<80% in patients treated with ART- vs TAF-based regimens).

**A. Year of inclusion**	**2017**	**2018**	**2019**
Patients, n	43	639	479
ART prescriptions	8.0 ± 2.4	7.9 ± 2.5	7.7 ± 2.4
HIV hospitalizations	0.2 ± 0.5	0.1 ± 0.3	0.1 ± 0.4
Number of other drugs	4.4 ± 5.0	3.8 ± 3.9	3.4 ± 4.0
Other hospitalizations	0.1 ± 0.5	0.1 ± 0.4	0.1 ± 0.4
Specialistic visits/diagnostic tests	8.5 ± 7.6	6.7 ± 6.7	5.9 ± 7.6

**B. Persistence**	**Non-persistent**	**Persistent**
Patients, n	256	939
ART prescriptions	8.0 ± 3.1	7.7 ± 2.3
HIV hospitalizations	0.1 ± 0.5	0.1 ± 0.3
Number of other drugs	4.3 ± 4.3	3.4 ± 3.9

Other hospitalizations	0.2 ± 0.5	0.1 ± 0.4
Specialistic visits/diagnostic tests	8.1 ± 8.4	6.1 ± 6.8

**C. Adherence**	**<80%**	**81-95%**	**>95%**
Patients, n	100	252	843
ART prescriptions	5.3 ± 2.4	7.7 ± 2.5	8.0 ± 2.4
HIV hospitalizations	0.1 ± 0.6	0.1 ± 0.3	0.1 ± 0.3
Number of other drugs	3.3 ± 4.4	3.5 ± 4.1	3.6 ± 3.9
Other hospitalizations	0.2 ± 0.6	0.1 ± 0.5	0.1 ± 0.4
Specialistic visits/diagnostic tests	5.3 ± 7.0	6.4 ± 7.7	6.7 ± 7.1

**D. Non-adherence (PDC <80%)**	**ART, adherence <80%**	**TAF, adherence <80%**
Patients, n	321	100
ART prescriptions	6.3 ± 3.7	5.7 ± 2.5
HIV hospitalizations	0.1 ± 0.2	0.1 ± 0.2
Number of other drugs	2.1 ± 1.9	2.3 ± 2.2
Other hospitalizations	0.1 ± 0.3	0.1 ± 0.3
Specialistic visits/diagnostic tests	6.1 ± 10.0	5.8 ± 7.0

Data are reported as mean ± standard deviation.

Table S3. Generalized linear model (GLM) for predictors of mean annual non-ART healthcare costs (€) during first year of follow up for patients with TAF-based regimens.

	**β (€)**	**95%CI**		**P value***
**Age 35**	**REF.**			
Age 35-50 years	415.3	-291.3	1,121.8	0.249
Age 51-65 years	1,363.6	557.5	2,169.6	**0.001**
Age >65 years	2,265.2	-260.6	4,791.0	0.079
Male gender	-62.0	-675.1	551.2	0.843
**Year 2017**	**REF.**			
Year 2018	-1,229.3	-4,141.3	1,682.7	0.408
Year 2019	-1829.8	-4,731.9	1,072.3	0.217
CCI	1,733.8	195.7	3,271.8	**0.027**
**Adherence <80%**	**REF.**			
Adherence 81-95%	-175.1	-1,574.1	1,223.9	0.806
Adherence >95%	-664.3	-1,913.6	585.0	0.297
Constant	3,373.9	81.1	6,666.7	0.045

CCI, Charlson Comorbidity Index.

* Significant P values are in bold.
